# Habitual Tea Consumption Is Associated with a Low Prevalence of Self-Reported Lifetime History of Depression in Taiwanese Population Study

**DOI:** 10.3390/nu18050841

**Published:** 2026-03-05

**Authors:** Si-Meng Chang, Jiun-Hung Geng, Pei-Yu Wu, Jiun-Chi Huang, Szu-Chia Chen

**Affiliations:** 1 Department of Medicine, Kaohsiung Medical University, Kaohsiung 807, Taiwan; u109001041@gap.kmu.edu.tw; 2 Department of Urology, Kaohsiung Medical University Hospital, Kaohsiung Medical University, Kaohsiung 807, Taiwan; u9001090@hotmail.com; 3 Department of Urology, Kaohsiung Municipal Siaogang Hospital, Kaohsiung Medical University Hospital, Kaohsiung Medical University, Kaohsiung 812, Taiwan; 4 School of Medicine, College of Medicine, Kaohsiung Medical University, Kaohsiung 807, Taiwan; wpuw17@gmail.com (P.-Y.W.); karajan77@gmail.com (J.-C.H.); 5 Department of Internal Medicine, Kaohsiung Municipal Siaogang Hospital, Kaohsiung Medical University Hospital, Kaohsiung Medical University, Kaohsiung 812, Taiwan; 6 Division of Nephrology, Department of Internal Medicine, Kaohsiung Medical University Hospital, Kaohsiung Medical University, Kaohsiung 807, Taiwan

**Keywords:** tea consumption, content, frequency and daily intake, self-reported lifetime history of depression, subgroup, Taiwan Biobank

## Abstract

Background/Objectives: Depression is a common mental disorder that has a substantial impact on both society and health. The potential health benefits of tea consumption have been suggested; however, whether the type of tea and drinking patterns such as frequency and intake are related to the risk of depression remain unclear, especially in different populations. This study utilized data from 27,119 Taiwan Biobank enrollees to evaluate the relationship between the prevalence of self-reported lifetime history of depression and tea consumption, including the type, drinking frequency, and daily intake. Methods: Tea consumption was categorized by type (fully fermented, semi-fermented, and non-fermented), frequency and daily intake. Self-reported questionnaires were used to record self-reported lifetime history of depression status. The association between tea consumption and self-reported lifetime history of depression was investigated using multivariable logistic regression. Results: Overall, tea consumption was significantly associated with a low prevalence of self-reported lifetime history of depression (odds ratio [OR] 0.736). While this association was found for semi-fermented and non-fermented teas (OR, 0.674), it was not found for fully fermented tea. Although a daily consumption of one–two cups (350–700 mL) was significantly associated with a low prevalence of self-reported lifetime history of depression, drinking more than three cups per day showed no association. Furthermore, regarding the frequency of tea consumption, those who drank tea every day were significantly associated with a low prevalence of self-reported lifetime history of depression, while those who only drank tea weekly or monthly were not. Subgroup analysis showed that tea consumption was not associated with a lower prevalence of self-reported lifetime history of depression in older individuals (≥65 years), those with diabetes, smokers, and those who drank alcohol, suggesting that health status and lifestyle factors may influence the possible associations of tea consumption. However, the interaction analysis did not achieve significance. We acknowledge that the formal interaction tests were not statistically significant and that these findings should therefore be considered exploratory. Conclusions: Consuming semi-fermented and non-fermented tea was associated with a low prevalence of self-reported lifetime history of depression; however, the association depended on the quantity and frequency of consumption. Further research is warranted to explore the biological mechanisms of different types of tea and develop intervention strategies for high-risk populations.

## 1. Introduction

Depression is a prevalent mental health disorder [1] characterized by impaired concentration and decision-making abilities, lack of interest, fatigue or lack of energy, a persistently low mood, and feelings of worthlessness or guilt [2]. It has a significant impact on both quality of life and society [3,4]. The lifetime prevalence of depression is estimated to be 12% [5], making it the 13th leading cause of disability and mortality worldwide [6], and the 4th leading cause of mortality in people 15 to 29 years of age [7]. The development of depression is influenced by a complex interplay of social, psychological and biological factors. Neurobiologically, depression has been linked to an imbalance of neurotransmitters in the brain [8,9], alterations in neuroplasticity and dysfunction in the connectivity between key brain regions [10,11]. Risk factors include genetic predisposition [12], environmental and social factors such as adverse childhood experiences [13], traumatic events [14], chronic stress [15], lack of social support [16], and unemployment [17], as well as general medical conditions such as endocrine and cardiovascular diseases [18,19]. Moreover, depression has also been linked to various comorbidities including cardiovascular disease [20] and metabolic syndrome [21]. Identifying tea consumption as a modifiable lifestyle factor may provide a foundation for future dietary intervention strategies to mitigate depression risk.

Tea is widely consumed globally, with potential health benefits including anti-aging [22], metabolic, anti-obesity [23], and antiviral [24] effects being extensively studied. Tea consumption has also been linked to potentially reduced risks of various diseases including diabetes [25], cardiovascular disease [26], liver disorders [27], and certain cancers, including prostate [28], oral [29], esophageal [30], nasopharyngeal [31], lung [32], ovarian [33] and bladder cancer [34]. Furthermore, animal and clinical studies have indicated a potential association between drinking tea and reduced depression risk [35], possibly mediated by the regulation of neurotransmitters through key bioactive compounds, including polyphenols and theanine [36,37]. However, these interpretations are primarily based on preclinical evidence, and no direct biomarkers were assessed in this study to support these mechanistic pathways. Therefore, the proposed biological mechanisms should be viewed as plausible but speculative. However, the existing literature on the impact of different types of tea, including fully fermented, semi-fermented, and non-fermented varieties, as well as drinking patterns such as frequency and daily intake, on depression remains limited. Fully fermented teas typically include black tea; semi-fermented teas are represented by oolong tea; and non-fermented teas include green tea and white tea. In particular, little research has explored these associations across different demographic characteristics such as age, sex, and chronic disease status.

This study utilized data from 27,119 Taiwan Biobank (TWB) enrollees and evaluated the relationship between the prevalence of self-reported lifetime history of depression and tea consumption, including the type, drinking frequency, and daily intake. We hypothesized that tea consumption may be associated with a lower prevalence of self-reported lifetime history of depression and aimed to determine whether this association varied across different demographic groups. The findings of this study may help elucidate the association between tea consumption and mental health, and may contribute to the development of future intervention strategies.

## 2. Materials and Methods

### 2.1. Identification of the Study Cohort

**Figure 1:**
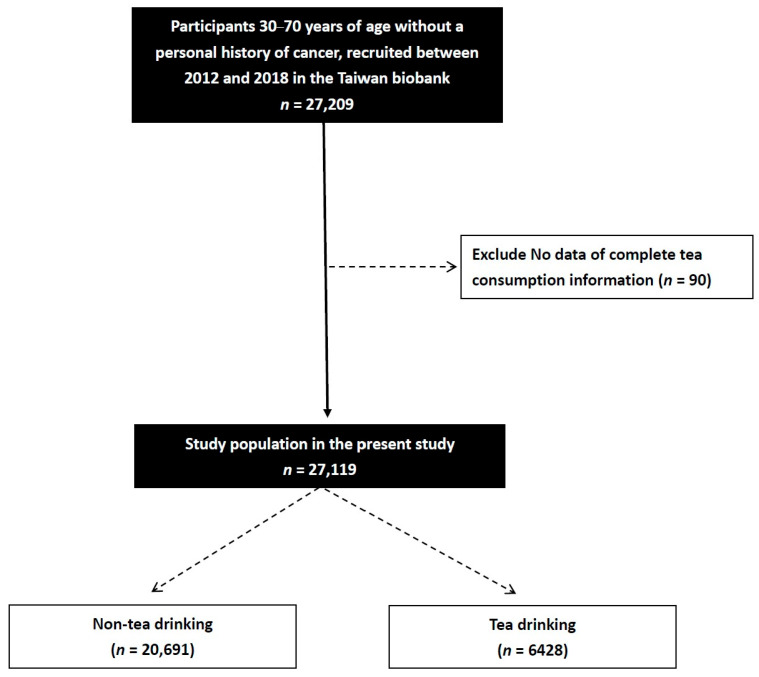
Flowchart of the study population.

Of the 27,209 participants in the TWB, 90 lacked information on tea consumption and were consequently not included in this study. The study cohort therefore included 27,119 participants (mean age 55.0 ± 10.3 years; 9589 males; 17,530 females) (Figure 1).

Managed by the Ministry of Health and Welfare, the TWB includes medical, genetic, and lifestyle information on Taiwanese adults 30–70 years of age with no previous diagnosis of cancer [38,39]. The aim of the TWB is to strengthen healthcare in Taiwan, considering ongoing challenges related to chronic diseases and the aging population. The Ethics and Governance Council of the TWB and Institutional Review Board (IRB) on Biomedical Science Research at Academia Sinica both oversee the TWB.

After obtaining written informed consent from each participant, data were collected through interviews, physical examinations, and blood tests. The collected data encompass histories of tobacco and alcohol consumption, the presence of diabetes mellitus [DM] and hypertension, sex, age, height and weight. Laboratory data include fasting total, high- (HDL) and low-density lipoprotein (LDL) cholesterol, glucose, uric acid, hemoglobin, and triglycerides. Estimated glomerular filtration rate (eGFR) is calculated using the 2021 Chronic Kidney Disease Epidemiology Collaboration creatinine equation [40].

Data on blood pressure (BP) were obtained through measurements made by trained personnel, and the averages of three systolic and diastolic BP measurements taken 1–2 min apart were recorded. All measurements were made after abstaining from caffeine, physical exercise, and smoking for a minimum of 30 min. In addition, the physical activity of the participants was assessed, with regular exercise defined as a minimum of three sessions of 30 min in 1 week [41]. This study complied with the principles established in the Declaration of Helsinki, and the Kaohsiung Medical University Hospital IRB granted ethical approval (KMUHIRB-E(I)-20210058, 23 March 2023).

### 2.2. Assessment of Tea Consumption

The study cohort was divided into habitual tea drinker and non-habitual tea drinker groups according to whether or not they drank tea regularly. In the habitual tea drinker group, information on the type of tea, daily intake, and frequency was obtained by asking the following questions:

1. “What type of tea do you usually drink?” Based on their answer, they were further classified as “fully fermented”, “semi-fermented” or “non-fermented” tea drinkers.
2. “How many cups of tea (one cup = 350 mL) do you usually drink per day?” Based on their answer, their daily intake was classified into “none”, “one”, “two” or “three or more” cups per day subgroups.
3. “How often do you drink tea (frequency)?” Based on their answer, they were classified into “never”, “daily”, “weekly” (frequency less than daily), and “monthly” (frequency less than weekly) frequency subgroups.

### 2.3. Self-Reported Lifetime History of Depression

The participants were asked during the interview “Have you ever had depression?” and then assigned to the self-reported lifetime history of depression or non-self-reported lifetime history of depression group accordingly.

### 2.4. Covariates

Covariates were selected based on prior epidemiological evidence and biological plausibility rather than solely on univariable statistical significance. Demographic factors (age, sex, marital status, and education) [42], lifestyle behaviors (smoking, alcohol consumption, tea and coffee intake, and regular exercise) [43], cardiometabolic conditions (diabetes mellitus, hypertension, body mass index, and systolic blood pressure) [44], and laboratory indicators of metabolic and renal function [45] were included because they have been consistently associated with both depression risk and health-related behaviors in population-based studies.

### 2.5. Statistical Analysis

Data are shown as number of participants with percentage or mean ± standard deviation. Differences between continuous and categorical data were assessed with independent t and chi-square tests, respectively. Associations among tea consumption, type of tea, intake and frequency with self-reported lifetime history of depression were evaluated in multivariable logistic regression analysis, which included age, sex, diabetes, hypertension, smoking and alcohol history, coffee consumption, regular exercise habit, married status, education status, systolic BP, body mass index, fasting glucose, hemoglobin, triglyceride, total cholesterol, eGFR and uric acid. We further conducted sensitivity analyses to provide further insights into these associations across different subgroups. Interactions among tea consumption on self-reported lifetime history of depression were examined using a logistic model, an interaction *p* in analysis: model disease (y) = x1 + x2 + x1 × x2 + covariates, where x1 × x2 is the interaction term; y = self-reported lifetime history of depression; x1 is tea consumption, and x2 is subgroup; and covariates = age, sex, diabetes, hypertension, smoking and alcohol history, coffee consumption, regular exercise habit, married status, education status, systolic BP, body mass index, fasting glucose, hemoglobin, triglyceride, total cholesterol, eGFR and uric acid (as above mentioned covariates in the methods: covariates were selected based on prior epidemiological evidence and biological plausibility). A *p* value < 0.05 was considered statistically significant. Statistical analysis was performed using SPSS version 25 (IBM Inc., Armonk, NY, USA).

## 3. Results

### 3.1. Comparison of Clinical Characteristics Between the Participants with and Without Self-Reported Lifetime History of Depression

Among the 27,119 participants, 1122 (4.1%) had self-reported lifetime history of depression and 25,997 (95.9%) did not (non-self-reported lifetime history of depression group). As shown in Table 1, those with self-reported lifetime history of depression were older, more likely to be female, had a higher prevalence of DM, lower rate of tea drinking, lower education status, lower systolic and diastolic BPs, hemoglobin, eGFR and uric acid, and higher total and HDL-cholesterol compared with those without self-reported lifetime history of depression.

### 3.2. Association Between Tea Consumption and Self-Reported Lifetime History of Depression

After age and sex adjustment, non-habitual tea drinkers (odds ratio [OR], 0.769; 95% confidence interval [CI], 0.657–0.899; *p* = 0.001) were associated with a high prevalence of self-reported lifetime history of depression. Multivariable logistic analysis was performed with adjustment for age, sex, diabetes, hypertension, smoking and alcohol history, coffee consumption, regular exercise habit, married status, education status, systolic BP, body mass index, fasting glucose, hemoglobin, triglyceride, total cholesterol, eGFR and uric acid (as above mentioned covariates in the methods: covariates were selected based on prior epidemiological evidence and biological plausibility). The results showed significant associations between older age (*p* < 0.001), female sex (*p* < 0.001), DM (*p* = 0.043), smoking (*p* < 0.001), non-habitual tea drinkers (OR, 0.736; 95% CI, 0.628–0.862; *p* = 0.001), never married (*p* = 0.012), low systolic BP (*p* < 0.001), high triglyceride (*p* = 0.030), high hemoglobin (*p* = 0.030), and low eGFR (*p* = 0.043), with a high prevalence of self-reported lifetime history of depression (Table 2).

### 3.3. Associations Among the Type of Tea, Intake and Frequency of Tea Consumption with Self-Reported Lifetime History of Depression

Multivariable logistic regression analysis was performed with adjustments for age, sex, diabetes, hypertension, smoking and alcohol history, coffee consumption, regular exercise habit, married status, education status, systolic BP, body mass index, fasting glucose, hemoglobin, triglyceride, total cholesterol, eGFR and uric acid (as above mentioned covariates in the methods: covariates were selected based on prior epidemiological evidence and biological plausibility) to assess the associations among the type of tea, intake and frequency of tea consumption with self-reported lifetime history of depression (Table 3). The results showed that those who drank semi- or non-fermented tea (OR, 0.674; 95% CI, 0.566–0.803; *p* < 0.001) were significantly associated with a low prevalence of self-reported lifetime history of depression. However, those who drank fully fermented tea (*p* = 0.577) were not significantly associated with a low prevalence of self-reported lifetime history of depression.

Table 3 also presents the ORs for self-reported lifetime history of depression based on the frequency of tea consumption among the study participants. Compared with the non-habitual tea drinkers, those who drank one cup daily (OR, 0.665; 95% CI, 0.509–0.869; *p* = 0.003) and those who drank two cups daily (OR, 0.668; 95% CI, 0.516–0.864; *p* = 0.002) had a significantly lower prevalence of self-reported lifetime history of depression. However, those who drank three cups or more daily (*p* = 0.352) were not significantly associated with a low prevalence of self-reported lifetime history of depression.

Regarding the frequency of tea consumption, compared with the non-habitual tea drinkers, those who consumed tea daily (OR, 0.710; 95% CI, 0.601–0.839; *p* < 0.001) were significantly associated with a low prevalence of self-reported lifetime history of depression. However, those who drank tea weekly (*p* = 0.921) and monthly (*p* = 0.175) were not significantly associated with self-reported lifetime history of depression.

### 3.4. Associations Among Tea Consumption and Self-Reported Lifetime History of Depression in Subgroup Analyses

To adjust for baseline differences between the habitual tea drinkers and non-habitual tea drinkers, we conducted subgroup analyses to further evaluate associations among tea consumption and self-reported lifetime history of depression (Table 4). Among those aged younger than 65 years, habitual tea drinkers had a lower prevalence of self-reported lifetime history of depression compared with non-habitual tea drinkers (OR, 0.728; 95% CI, 0.609–0.870; *p* < 0.001). In addition, male (OR: 0.675, 95% CI [0.507–0.898]; *p* = 0.007) and female (OR: 0.776, 95% CI [0.641–0.939]; *p* = 0.009) habitual tea drinkers also had a lower prevalence of self-reported lifetime history of depression compared with non-habitual tea drinkers. Among the participants without DM, habitual tea drinkers had a lower prevalence of self-reported lifetime history of depression compared with non-habitual tea drinkers (OR, 0.742; 95% CI, 0.628–0.878; *p* = 0.001). In both the participants with (OR: 0.671, 95% CI [0.470–0.960]; *p* = 0.029) and without hypertension (OR: 0.753, 95% CI [0.630–0.899]; *p* = 0.002), habitual tea drinkers had a lower prevalence of self-reported lifetime history of depression compared with non-habitual tea drinkers. In non-smokers, drinking tea was associated with a slower prevalence of self-reported lifetime history of depression (OR: 0.712, 95% CI [0.584–0.867]; *p* = 0.001), and a lower prevalence of self-reported lifetime history of depression was also found in those who did not consume alcohol (OR: 0.729, 95% CI [0.614–0.866]; *p* < 0.001). In both participants who did (OR: 0.741, 95% CI [0.591–0.930]; *p* = 0.010) and did not exercise regularly (OR: 0.735, 95% CI [0.588–0.919]; *p* = 0.007), habitual tea drinkers had a lower prevalence of self-reported lifetime history of depression compared with non-habitual tea drinkers. However, those aged older than 65 years, those with DM, those who had ever smoked, and those who drank alcohol were not associated with a lower prevalence of self-reported lifetime history of depression. However, the interaction analysis between tea consumption and all variables on depression did not achieve significance.

## 4. Discussion

The results of this study demonstrated that habitual tea drinkers were associated with a significantly lower prevalence of self-reported lifetime history of depression, with the most pronounced associations with semi- and non-fermented teas. Drinking one to two cups of tea (approximately 350–700 mL) a day was significantly associated with a lower prevalence of self-reported lifetime history of depression, while a higher intake did not provide a significant relationship. Tea consumption frequency was also an important factor, as only those who drank tea daily had a significantly lower prevalence of self-reported lifetime history of depression, whereas drinking tea weekly or monthly showed no significant association. Subgroup analysis showed that tea consumption was not associated with a lower prevalence of self-reported lifetime history of depression in older individuals (≥65 years), those with DM, smokers, and those who drank alcohol, suggesting that health status and lifestyle factors may influence the possible associations of tea consumption. However, the interaction analysis did not achieve significance.

The first important finding is the association between drinking semi- and non-fermented teas with a low prevalence of self-reported lifetime history of depression. A previous study in China analyzed 7524 adults aged 25 to 90 years and reported that those who drank green tea (non-fermented tea) daily were associated with a lower risk of depression (hazard ratio = 0.78, 95% CI: 0.63–0.97) [46]. In addition, the China Longitudinal Healthy Longevity Survey, which included 13,115 individuals aged 65 years and older, found that regular consumption of black (fermented tea), oolong (semi-fermented tea) and green teas was associated with a lower risk of depression, with green tea showing the most pronounced protective effect [47]. The potential biological mechanisms underlying the association between consuming tea and reduced depression risk may involve the regulatory effects of polyphenols and theanine on the nervous system [36,37]. Cellular and animal studies have shown that polyphenols and theanine possess antioxidant and anti-inflammatory properties that can help mitigate chronic inflammation-induced neuronal damage [48,49]. In addition, theanine has been suggested to inhibit the reuptake of gamma-aminobutyric acid [50] and modulate serotonin and dopamine levels in specific brain regions [51], thereby alleviating symptoms of depression and anxiety [52]. However, given the cross-sectional design of the present study, causal inferences cannot be established, and the possibility of reverse causation should be considered. It is plausible that individuals with depressive symptoms may alter their tea consumption habits due to changes in mood, sleep patterns, appetite, or overall lifestyle rather than tea consumption directly influencing depression risk. Consequently, the observed associations should be interpreted with caution, and prospective longitudinal studies are needed to clarify the temporal relationship between tea consumption and depression. However, in this study, fully fermented tea did not have a significant association, possibly due to the reduction of active compounds during the fermentation process attenuating its potential health benefits [53]. Further investigations are warranted to elucidate these underlying mechanisms. In addition, it is important to consider the distribution of participants across different tea types, as fully fermented teas may have been consumed by a relatively smaller or distinct subgroup, potentially limiting statistical power. Differences in consumption patterns, such as frequency and portion size, could also contribute to the observed lack of association. Furthermore, the degree of fermentation was categorized based on general tea types, which may not fully capture individual variability or preparation methods, introducing potential exposure misclassification. These factors warrant cautious interpretation and highlight the need for more detailed data in future studies.

Our results also indicate that the quantity of tea intake may be associated with self-reported lifetime history of depression. In this study, participants who drank one to two cups of tea daily exhibited significant associations, whereas no association was found in those who consumed three or more cups daily. A study on 537 Japanese workers aged 20 to 68 years reported an association between drinking two or more cups of green tea daily with a lower risk of depression (OR = 0.61, 95% CI: 0.38–0.98) [54]. The lack of significant association in our participants who drank three or more cups of tea per daily may be attributed to the possible adverse effects of high tea consumption, including caffeine- or tannin-related side effects such as insomnia, anxiety, or gastrointestinal discomfort [55]. Further studies are warranted to further clarify the threshold between adequate and excessive tea consumption and the effect on mental health. In addition to the quantity of tea consumed, we also found that frequency was an important factor. A significant association was only found in daily tea drinkers, whereas this association was not found in those who consumed tea weekly or monthly. This is in line with a previous study on 1368 Chinese individuals aged ≥ 60 years, which reported that daily tea drinkers had a significantly lower risk of depression (OR = 0.59, 95% CI: 0.43–0.81), while those who drank tea weekly did not have a significantly lower risk [56]. Another study conducted in China reported that drinking one or more cup of tea daily was significantly associated with a lower risk of depression. In addition, a linear relationship was observed between the risk of depression and frequency of tea consumption, indicating that a lower risk of depression was associated with a higher frequency of tea consumption [47].

An interesting finding of the present study is that the associations of tea consumption with self-reported lifetime history of depression were not significant in certain subgroups, particularly older individuals (≥65 years), those with diabetes, smokers, and those who drank alcohol. However, the interaction analysis did not achieve significance. These groups are generally characterized by increased levels of chronic inflammation, oxidative stress, and neurotransmitter dysregulation, which may attenuate the neuroprotective effects of tea polyphenols and theanine [57,58,59,60]. In addition, an association has been reported between alcohol consumption with long-term structural and functional changes in the brain, potentially further diminishing the benefits of tea [61]. Moreover, age-related declines in metabolism and nutrient absorption may affect the bioavailability and efficacy of the active compounds in tea [62]. These findings suggest that specific health conditions and lifestyle factors may modulate the association between tea consumption and mental health. Although the protective association of tea consumption was not statistically significant in certain subgroups, such as individuals with chronic diseases or lifestyle risk factors, the direction of association generally remained consistent across most strata, suggesting a potentially universal benefit. This pattern may imply that the bioactive compounds in tea exert baseline neuroprotective effects that are not easily overridden by variations in individual health or behavior. Alternatively, it is possible that residual confounding or limited statistical power in the subgroup analyses obscured more nuanced interactions. Future studies with larger samples and stratified designs are needed to validate these findings and clarify whether the observed consistency reflects true biological robustness or methodological limitations. However, the non-significant relationships of subgroup analyses may stem from diminished statistical power rather than a genuine lack of relationship. We acknowledge that the formal interaction tests were not statistically significant and that these findings should therefore be considered exploratory. Further research into the underlying mechanisms is warranted.

Although this study provides novel insights into the association between tea consumption and self-reported lifetime history of depression, the cultural and demographic context must be considered when interpreting the findings. The study cohort consisted primarily of Taiwanese individuals, whose tea-drinking behaviors are shaped by long-standing cultural traditions, and whose genetic and healthcare backgrounds may differ from those of Western or non-Asian populations. These differences may limit the external validity and generalizability of our findings across diverse ethnic and sociocultural settings.

Our findings are strengthened by the comprehensive analysis of a large community-based population of healthy individuals. However, there are also several limitations. First, depression status was assessed using a single retrospective self-reported item (“Have you ever had depression?”), which reflects participants’ lifetime history of depression rather than current symptom severity. This approach does not constitute a standardized psychiatric diagnostic interview or a fully validated symptom-based scale, such as the nine-item Patient Health Questionnaire or the Center for Epidemiologic Studies Depression Scale. Although brief standardized screening instruments, including the two-item Patient Health Questionnaire (PHQ-2), are available within the TWB, these data were collected only in a subset of participants and were therefore insufficient for use as the primary outcome definition in the present study. Consequently, some degree of outcome misclassification cannot be excluded. Importantly, however, a validation study conducted by Academia Sinica, the institution responsible for administering the TWB, demonstrated that self-reported psychiatric disorders, including major depressive disorder, showed substantial concordance with National Health Insurance claims data, with a tetrachoric correlation of 0.758 [63]. These findings support the reasonable predictive validity of self-reported depression measures within the Taiwanese population and partially mitigate concerns regarding outcome misclassification. Second, due to the cross-sectional design of this study, we could not analyze the duration of depression or causal relationship between depression and tea consumption. Longitudinal studies are needed to investigate the likelihood of developing new-onset depression. Third, we did not determine the maximum tea intake associated with depression, because caffeine and tannin-related side effects were possible. Fourth, although classifying the type of tea by degree of fermentation is common, subtle differences remain regarding the tea plant species and place of origin. Fifth, we lack the information on socioeconomic position, sleep quality, and the dietary patterns of participants, as these data can significantly influence both tea consumption habits and depression prevalence. Further research is needed to clarify the influence between the information and tea consumption and its effects on mental health. Sixth, the absence of further association among participants consuming more than three cups of tea per day may indicate a plateau association rather than a true lack of biological benefit. Notably, the number of individuals reporting high levels of tea consumption was relatively small, which may have limited statistical power and resulted in less precise estimates with wider confidence intervals. Consequently, association estimates in the highest intake category may be more susceptible to instability and the influence of extreme values. Therefore, the observed dose–response pattern should be interpreted cautiously, and future studies with a broader distribution of high-level tea consumption are needed to more definitively evaluate potential nonlinear associations. Finally, more women than men were enrolled in this study, possibly because women are generally more willing to volunteer for research studies, and this may have affected the generalizability of our findings to male individuals. In addition, most of the study cohort were Taiwanese, which may limit the generalizability of our findings to other groups with different environmental, cultural, or genetic backgrounds.

## 5. Conclusions

This study provides evidence supporting an association between tea consumption and a low prevalence of self-reported lifetime history of depression, and particularly the daily consumption of moderate amounts of semi-fermented and non-fermented teas. However, due to the cross-sectional design of this study, the causal relationship between tea consumption and self-reported lifetime history of depression could not be confirmed. Longitudinal studies are needed to investigate the likelihood of developing new-onset depression. Furthermore, the definition of depression in the TWB is too simplistic, and further research is warranted to explore potential influencing factors and clarify the relationship between tea consumption and mental health outcomes across different populations, using standardized depression scales or clinical diagnosis by a physician.

## Figures and Tables

**Table 1:** Comparisons of clinical characteristics between the participants with and without self-reported lifetime history of depression.

Characteristics	Self-Reported Lifetime History of Depression (−) (*n* = 25,997)	Self-Reported Lifetime History of Depression (+) (*n* = 1122)	*p*
Age (year)	55.0 ± 10.3	56.4 ± 9.7	<0.001
Male sex (%)	35.9	23.9	<0.001
Diabetes mellitus (%)	7.6	9.4	0.034
Hypertension (%)	17.8	19.5	0.143
Smoking history (%)	25.6	25.6	0.986
Alcohol history (%)	10.7	9.8	0.352
Habitual tea drinkers (%)	23.9	18.0	<0.001
Coffee consumption (%)	41.2	40.9	0.858
Regular exercise habits (%)	48.2	49.6	0.352
Ever married (%)	92.6	91.4	0.152
Education status			<0.001
Elementary school and below (%)	7.3	7.8	
Junior/senior high school (%)	43.7	49.3	
University or above (%)	49.0	43.0	
Systolic BP (mmHg)	124.9 ± 19.5	122.0 ± 18.2	<0.001
Diastolic BP (mmHg)	74.3 ± 11.4	72.9 ± 10.9	<0.001
Body mass index (kg/m^2^)	24.3 ± 3.6	24.2 ± 3.8	0.273
Laboratory parameters			
Fasting glucose (mg/dL)	97.4 ± 21.8	97.5 ± 20.9	0.935
Hemoglobin (g/dL)	13.7 ± 1.6	13.6 ± 1.4	0.003
Triglyceride (mg/dL)	119.9 ± 97.1	125.1 ± 93.3	0.074
Total cholesterol (mg/dL)	196.1 ± 36.2	199.3 ± 35.7	0.003
HDL-C (mg/dL)	54.5 ± 13.5	55.3 ± 13.4	0.049
LDL-C (mg/dL)	120.1 ± 31.9	121.8 ± 31.8	0.086
eGFR (mL/min/1.73 m^2^)	98.0 ± 15.1	97.0 ± 15.0	0.021
Uric acid (mg/dL)	5.4 ± 1.4	5.3 ± 1.3	0.001

Abbreviations. BP, blood pressure; eGFR, estimated glomerular filtration rate; HDL-C, high-density lipoprotein cholesterol; LDL-C, low-density lipoprotein cholesterol.

**Table 2:** Association between tea consumption with self-reported lifetime history of depression in multivariable regression analysis.

Variables	Multivariable	
	OR (95% CI)	*p*
Age (per 1 year)	1.018 (1.009–1.027)	<0.001
Male sex (vs. female)	0.400 (0.323–0.496)	<0.001
Diabetes mellitus	1.295 (1.009–1.663)	0.043
Hypertension	1.183 (0.994–1.407)	0.059
Smoking history	1.700 (1.421–2.032)	<0.001
Alcohol history	1.115 (0.892–1.393)	0.339
Habitual tea drinkers	0.736 (0.628–0.862)	<0.001
Coffee consumption	0.990 (0.875–1.121)	0.876
Regular exercise habits	0.989 (0.871–1.125)	0.872
Ever married	0.751 (0.600–0.939)	0.012
Education status		
Elementary school and below	Reference	
Junior/senior high school	1.212 (0.955–1.539)	0.113
University or above	1.129 (0.878–1.451)	0.345
Systolic BP (per 1 mmHg)	0.988 (0.985–0.992)	<0.001
Body mass index (per 1 kg/m^2^)	1.010 (0.991–1.029)	0.316
Laboratory parameters		
Fasting glucose (per 1 mg/dL)	0.998 (0.994–1.001)	0.231
Hemoglobin (per 1 g/dL)	1.058 (1.005–1.114)	0.030
Triglyceride (per 1 mg/dL)	1.001 (1.000–1.001)	0.030
Total cholesterol (per 1 mg/dL)	1.001 (1.000–1.003)	0.149
eGFR (per 1 mL/min/1.73 m^2^)	0.994 (0.989–1.000)	0.043
Uric acid (per 1 mg/dL)	0.976 (0.923–1.032)	0.388

See Table 1 for the abbreviations. Values are expressed as odds ratio (OR) and 95% confidence interval (CI). Adjusted for age, sex, diabetes, hypertension, smoking and alcohol history, coffee consumption, regular exercise habit, married status, education status, systolic BP, body mass index, fasting glucose, hemoglobin, triglyceride, total cholesterol, eGFR and uric acid (as above mentioned covariates in the methods: covariates were selected based on prior epidemiological evidence and biological plausibility).

**Table 3:** Associations among type, daily intake and frequency of tea consumption with self-reported lifetime history of depression in multivariable regression analysis.

Tea Consumption (Number of Self-Reported Lifetime History of Depression Cases/Subgroup Number)	Multivariable	
	OR (95% CI)	*p*
Types of tea		
Non-tea (920/20,691)	Reference	
Fully fermented tea (43/950)	1.094 (0.798–1.500)	0.577
Semi- or non-fermented tea (159/5478)	0.674 (0.566–0.803)	<0.001
Daily cups		
None (690/20,691)	Reference	
1 cup * per day (60/2030)	0.665 (0.509–0.869)	0.003
2 cups * per day (65/2312)	0.668 (0.516–0.864)	0.002
≥3 cups * per day (77/2086)	0.891 (0.699–1.136)	0.352
Frequency		
None (920/20,691)	Reference	
Per day (180/5931)	0.710 (0.601–0.839)	<0.001
Per week (20/477)	0.977 (0.620–1.541)	0.921
Per month (2/20)	2.784 (0.635–12.218)	0.175

See Table 1 for the abbreviations. Adjusted for age, sex, diabetes, hypertension, smoking and alcohol history, coffee consumption, regular exercise habit, married status, education status, systolic BP, body mass index, fasting glucose, hemoglobin, triglyceride, total cholesterol, eGFR and uric acid (as above mentioned covariates in the methods: covariates were selected based on prior epidemiological evidence and biological plausibility). * One cup = 350 mL.

**Table 4:** Subgroup analyses: association between tea consumption and self-reported lifetime history of depression.

Subgroups (Number of Self-Reported Lifetime History of Depression Cases/Subgroup Number)	Self-Reported Lifetime History of Depression OR (95% CI)	*p*	Interaction *p*
Age			0.885
<65 years (868/21,563)	0.728 (0.609–0.870)	<0.001	
≥65 years (254/5556)	0.764 (0.538–1.085)	0.133	
Sex			0.199
Male (268/9589)	0.675 (0.507–0.898)	0.007	
Female (854/17,530)	0.776 (0.641–0.939)	0.009	
Diabetes mellitus			0.703
Yes (105/2090)	0.703 (0.429–1.151)	0.161	
No (1017/25,029)	0.742 (0.628–0.878)	0.001	
Hypertension			0.576
Yes (219/4848)	0.671 (0.470–0.960)	0.029	
No (903/2227)	0.753 (0.630–0.899)	0.002	
Smoking			0.911
Yes (287/6943)	0.778 (0.594–1.019)	0.068	
No (835/20,176)	0.712 (0.584–0.867)	0.001	
Alcohol			0.962
Yes (110/2886)	0.784 (0.513–1.196)	0.259	
No (1012/24,233)	0.729 (0.614–0.866)	<0.001	
Regular exercise habits			0.881
Yes (557/13,094)	0.741 (0.591–0.930)	0.010	
No (565/14,025)	0.735 (0.588–0.919)	0.007	

See Table 1 for the abbreviations. Adjusted for age, sex, diabetes, hypertension, smoking and alcohol history, coffee consumption, regular exercise habit, married status, education status, systolic BP, body mass index, fasting glucose, hemoglobin, triglyceride, total cholesterol, eGFR and uric acid (as above mentioned covariates in the methods: covariates were selected based on prior epidemiological evidence and biological plausibility).

## Data Availability

The data underlying this study are from the Taiwan Biobank. Due to restrictions placed on the data by the Personal Information Protection Act of Taiwan, the minimal data set cannot be made publicly available. Data may be available upon request to interested researchers. Please send data requests to: Szu-Chia Chen. Division of Nephrology, Department of Internal Medicine, Kaohsiung Medical University Hospital, Kaohsiung Medical University.
